# Ultrafast Permittivity Engineering Enables Broadband Enhancement and Spatial Emission Control of Harmonic Generation in ZnO

**DOI:** 10.1021/acsphotonics.4c01737

**Published:** 2024-11-15

**Authors:** Zhonghui Nie, Kevin Murzyn, Leo Guery, Thomas J. van den Hooven, Peter M. Kraus

**Affiliations:** † Advanced Research Center for Nanolithography, Science Park 106, 1098 XG, Amsterdam, The Netherlands; ‡ Department of Physics and Astronomy, and LaserLaB, Vrije Universiteit, De Boelelaan 1105, 1081 HV Amsterdam, The Netherlands

**Keywords:** Nonlinear optics, high harmonic generation, epsilon near zero, permittivity engineering, ultrafast modulation, microscopy

## Abstract

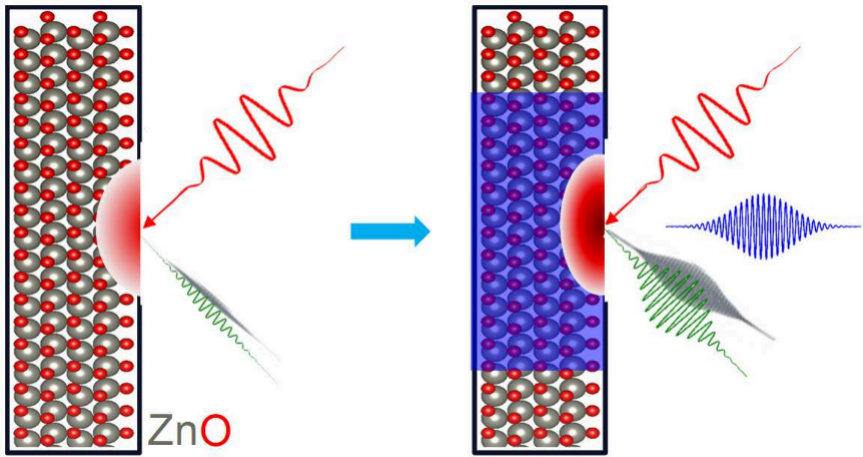

Moderate efficiencies of nonlinear optical processes can be one of the challenges limiting even more widespread applications. Here we demonstrate a broadband and giant enhancement of nonlinear processes in ZnO through ultrafast permittivity engineering. A remarkable enhancement of the second and third harmonic generation of up to 2 orders of magnitude can be observed over a broadband range of driving wavelengths. Moreover, this nonlinearity enhancement is reversible with a recovery time of ∼120 fs. Additional experiments and simulations confirm that the observed enhancement originates from a permittivity change induced by the photocarrier population. Our results provide the opportunity to actively customize materials with a larger nonlinearity for nanophotonics on ultrafast time scales over broadband wavelength ranges. Utilizing this finding, we also demonstrate a relevant application, where a transient wave-guiding effect is induced by a donut-shaped photocarrier-excitation pulse, which both reduces the width of the spatial profile of harmonic emission below the diffraction limit and simultaneously increases its central emission strength.

## Introduction

Nonlinear optical light–matter interactions are a powerful way for broadband light generation^1,2^ as well as for probing material properties via for instance harmonic generation and wave mixing.^3−9^ These applications almost always benefit from improving the nonlinear efficiency.^10−14^ For solid-state harmonic generation, one such avenue is via dielectric metasurfaces^15−17^ which enhances nonlinear efficiency due to periodic nanostructures that support Mie resonances. As a consequence, such metasurfaces work only for certain wavelengths, with a relatively low damage threshold. Another promising avenue is the modification of the dielectric function^18,19^ via for instance elemental doping. In certain cases, strong doping can bring the dielectric function close to zero (similar to metals, but with a smaller imaginary part, i.e. low absorption), which then gives rise to strong electric-field enhancement inside the material^20,21^ and therefore could in principle enhance nonlinear optical efficiency.^22−25^ While not necessarily applicable to all materials, this effect bares the great advantage that it is broadband. Photoexcitation can cause similar effects to doping and is thus sometimes called photodoping. In specific cases such as zinc oxide (ZnO), photodoping was found to transiently decrease the dielectric function close to zero^26^ which in principle could open the door for enhancing harmonic generation. However, the overall tenor for solid-state high-harmonic generation (HHG) has thus far been that photodoping suppresses HHG,^9,27−34^ whereas there are some exceptions that, e.g., report HHG boosting via enhanced intraband currents by photodoping.^35^ Importantly, HHG suppression was (among many other examples) also shown for ZnO.^36−38^

In this letter we set out to systematically investigate harmonic generation in photoexcited ZnO, the prime example of a material where photodoping can shift the real part of the dielectric function close to zero while keeping the imaginary part small, which is called the epsilon-near-zero (ENZ) effect.^20,26^ We show that for specific excitation fluences and driving-wavelength polarization, harmonic generation can in fact be enhanced by up to 2 orders of magnitude, whereas for other driving conditions, the previously observed HHG suppression^27,34,36−38^ is confirmed. We furthermore show how transient manipulation of the dielectric function allows engineering the point spread function (PSF, i.e., the spatial emission profile) to a width that is below the diffraction limit,^39^ while enhancing the central emission strength. During the preparation for manuscript submission, a recent submission on a similar topic came to our attention.^40^ Our results agree with and further elaborate on these very recently released findings on harmonic generation enhancement in ZnO.^40^

## Results and Discussion

**Figure 1:**
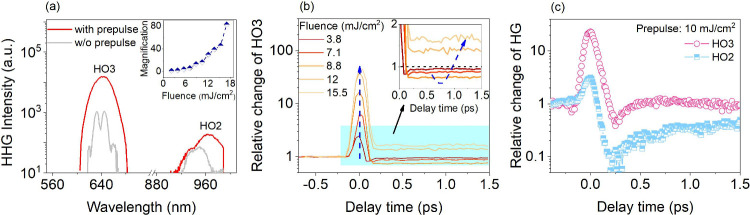
Giant enhancement of harmonic generation (HG) signals under the excitation of a prepulse (pump). (a) Direct comparison of the reflected HG spectra from ZnO with and without the prepulse (10.5 mJ/cm^2^) at time zero, where HO2 and HO3 are increased by more than 1 order of magnitude. The inset shows the enhancement of HO3 as a function of the prepulse fluence, where a nearly exponential increase can be observed. (b) Dynamics of HO3 under different prepulse fluences from 3.8 to 15.5 mJ/cm^2^. The inset shows a zoom-in of the main plot, highlighting a suppression of HG after pulse overlap at low to moderate fluences and an enhancement at high fluences. (c) Comparison of HO2 and HO3 dynamics, under the same experimental condition as (a).

Static harmonic generation (HG) reflected from single crystal ZnO (0001) (Surfacenet GmbH) is produced by a 70 fs and 1.95 μm driving pulse in reflection mode and collected by a fiber spectrometer (see details in Methods). Under the driving intensity of 0.7 TW/cm^2^, two harmonic orders (HO2 and HO3) are observed clearly from the ZnO surface, shown in Figure 1a. To test the transient response of HG, a prepulse (pump) with a wavelength of 400 nm is added to excite ZnO and the corresponding HG spectra are plotted in Figure 1a. We observe that a prepulse (10.5 mJ/cm^2^) can enhance all harmonic orders by more than 1 order of magnitude. Moreover, the increase in the prepulse fluence leads to a near-exponential amplification of the HO3 intensity by up to 2 orders of magnitude, as shown in the inset of Figure 1a. It should be noted that the prepulse fluence applied in the manuscript is within 20 mJ/cm^2^, and much below the damage threshold of ZnO, which is above 100 mJ/cm^2^. Figure 1b shows the transient responses of HO3 under several prepulse fluences. Here a signal of one means that the transient HO3 emission intensity is the same as that without the prepulse, and negative delays represent time delays where the prepulse arrives at the sample later than the HG driving pulse. The transient enhancement of up to 2 orders of magnitude at time zero lasts for a full-width half-maximum (fwhm) of 120 fs corresponding to the cross-correlation of both pulses. The HG enhancement at high prepulse fluences lasts for several picoseconds and overcompensates for HG suppression that dominates at low fluences, as seen in the inset of Figure 1b. Similar to that of HO3, a remarkable enhancement at time zero can also be observed in HO2, as shown in Figure 1c, but the subsequent dynamics of these two harmonics are slightly different. The even-order harmonics (such as HO2) are generated only from the ZnO surface and are more sensitive to the lattice symmetry, and transient lattice distortions induced by photocarriers could suppress and even cover the HG enhancement. HO3 as an odd-order harmonic is out of these limits and will be mainly investigated in the manuscript.

While a saturation effect might inevitably weaken the prepulse influence at higher fluences, the HG enhancement increases (possibly exponentially) with the prepulse fluence in our experiments (inset of Figure 1a). This enhancement seems to be an opposite finding compared to previous HG investigations in ZnO and other semiconductors,^27,36−38^ where HG suppression was nearly always observed. Suppression of HG is typically assigned to an increased scattering rate (i.e., decreased dephasing time) induced by photoexcited carriers. In fact, we did observe a long-lasting suppression (several hundred picoseconds) at low and moderate prepulse fluences at time delays after pump–probe overlap, as highlighted in the inset of Figure 1b. The observed time scale is similar to photocarrier lifetime in ZnO.^26^

**Figure 2:**
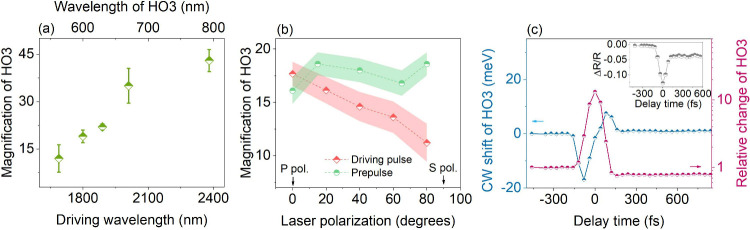
(a) Enhancement of HO3 for five distinct driving wavelengths under the same prepulse excitation of 12 mJ/cm^2^. The observed HG enhancement can be realized over a broad-band range of driving wavelengths. Longer wavelengths support a stronger enhancement. (b) The relationship between HO3 enhancement and the polarization of prepulse (green, HG driver has fixed p-polarization) and HG driving pulse (prepulse has fixed p-polarization). The shaded areas represent experimental error bars, calculated from multiple measurements, and the prepulse fluence of 9 mJ/cm^2^ is constant for all measurements. (c) Dynamics of HO3 intensity and its center wavelength change under the prepulse of 9 mJ/cm^2^. The large change in the center wavelength is indicative of a strong refractive index/permittivity variation induced by the prepulse excitation, and it temporally overlaps with transient HO3 enhancement. Furthermore, a rapid change in linear reflectivity of the driving wavelength (1.8 μm) as shown in the inset is also observed and points to the same conclusion of a strong refractive index/permittivity variation.

We now proceed to identify an optimum set of parameters that enable HG enhancement in ZnO at high prepulse fluences. The effect of the driving wavelength is first investigated to rule out possible resonant effects. Under the same prepulse fluence of 12 mJ/cm^2^ and parallel p-polarizations, we observe an enhancement of HO3 generated from five different driving wavelengths (from 1600 to 2400 nm) as shown in Figure 2a. Despite different levels of magnifications, i.e., the ratio between the enhanced and intrinsic HO3, the enhancement of HO3 can be observed for all tested driving wavelengths. The transient change of HO3 at these driving wavelengths also exhibits similar dynamics. This unambiguously demonstrates that the HG enhancement induced by the prepulse can be realized over a broadband range and overcomes one of the largest drawbacks of resonant nonlinear enhancement elements that are designed for certain narrowband wavelengths.

Next, several polarization combinations between the prepulse and driving beam were tested by rotating two independent broadband waveplates, and their influences on the HG enhancement are displayed in Figure 2b. Within experimental error bars, the HG enhancement is almost independent of the prepulse polarization (green points and shaded area in Figure 2b). This supports the hypothesis that the photocarrier population excited by the prepulse instead of the electric field of the prepulse is the origin of the HG enhancement. Interestingly, the HG enhancement displays a strong dependence on the polarization of the driving beam (red points and shaded area in Figure 2b), and the HG enhancement is reduced by half when tuning the polarization from p to s polarization. This unusually strong polarization dependence indicates a field enhancement of the driving pulse as a dominant mechanism for the observed HG increase.

In addition, the transient linear reflectivity of the driving beam (1.8 μm) is also monitored (inset in Figure 2c) and should be compared to the dynamics of HO3 (magenta and blue lines in Figure 2c showing the center-wavelength shift and relative change of HO3, respectively). The linear reflection suddenly reduces and partially recovers within 200 fs, and its time scale is identical within error bars to that observed in the HO3 dynamics (Figure 1b and magenta line in Figure 2c). A transient reflection change originates from the transient modification of the refractive index or permittivity, indicating that the HG enhancement may result from a transient change of permittivity, induced by photocarriers. In fact, a similar permittivity change through photodoping has been reported in thin film ZnO earlier,^26^ and strong excitation even could reduce the permittivity below zero, which is known as the epsilon near zero (ENZ) effect. We also show the change in the central wavelength during HG enhancement in Figure 2c (blue line). The central wavelength of HO3 first shifts to the red side before time zero, and then to the blue side after time zero. This profile resembles the typical wavelength variation during a transient phase shift of a laser pulse as present in self-phase modulation (or in this case: cross-phase modulation between two pulses), which originates from an intensity-dependent modification of the refractive index. The shift is particularly large compared to previously reported shifts of HG following photoexcitation.^9^ As a low overall permittivity generally leads to a large nonlinear refractive index, this observation further corroborates the HG enhancement due to the ENZ effect.^20^ It should be noted that the magnitude of the wavelength shift is not symmetric around time zero, but instead the transient red-shift is stronger than the transient blueshift. Moreover, after pump–probe overlap, there is a small but lasting blueshift. This indicates two competing effects at play: a transient modification of the refractive index, i.e., cross-phase modulation, and a lasting modification due to an excited carrier population. The contribution of the latter becomes dominant as soon as sufficient carriers are excited, which becomes apparent after time zero at higher fluences.

The combination of the experimental evidence from Figures 1 and 2 paints the following picture of ultrafast and broadband enhancement of HG in ZnO. After time zero (*t* > 200 fs), the carrier excitation at low and moderate fluences mainly creates HG suppression as previously observed (the inset of Figure 1b). At higher prepulse fluences, highly dense photocarriers lower the refractive index/dielectric function a lot such that a p-polarized electric field of the driving pulse gets enhanced and therefore HG is more efficient. When both pulses are synchronized around time zero, the combined effect of the prepulse and HG driving pulse leads to a strongly enhanced carrier injection rate, as band gap (3.4 eV) excitation becomes accessible via two-photon excitation with one photon each of the prepulse (400 nm, 3.1 eV) and HG driver (1700–2400 nm, 0.73–0.52 eV). Moreover, the strong light field of these two pulses can lead to a field-enhanced carrier injection around time zero.^41^ Therefore, the HG enhancement is strongest during the overlap of both pulses, where a large photocarrier fraction brings the dielectric function close to zero. Remarkably, this giant enhancement is reversible with a recovery time of ∼120 fs, as seen in Figure 1b and 2c. Such a short time means that the prepulse can be utilized not only for brighter HG sources but also as a nonlinear switch with a working frequency of nearly 10 THz and a high on–off ratio of 20 dB.

**Figure 3:**
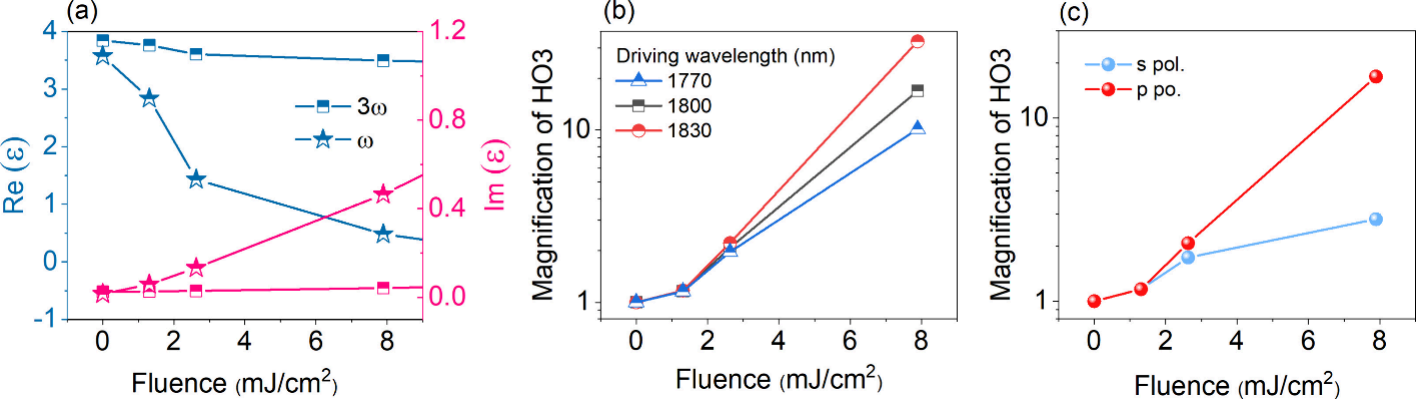
(a) Real and imaginary parts of permittivity of ZnO at the driving wavelength (1800 nm) and HO3 wavelength (600 nm) as a function of prepulse fluence, where photoinduced carriers alter the permittivity of ZnO. These values are extrapolated from the equation proposed in the reference.^26^ (b, c) Calculations (using the permittivity from (a)) of HO3 enhancement as a function of prepulse fluence for several driving wavelengths (b) and s- and p-polarization comparison (c), respectively.

Most of our findings can be reproduced through the simulation based on the above hypothesis of a transient permittivity reduction that enhances HG (details described in Methods section).^42,43^ Saha et al.^26^ carried out transient broadband reflectivity measurements under a series of prepulse fluences to extract the transient permittivity of ZnO. We extrapolated these measurements to our experimental parameters, and show the dielectric function of photoexcited ZnO at the driving and HO3 wavelengths (ω and 3ω in Figure 3a for denoting the respective angular frequencies of the fundamental and third harmonic). With the increase of the prepulse fluence (corresponding to an increase of photocarrier density), the real part of permittivity at both wavelengths with angular frequencies ω and 3ω reduces. However, the effect is far more significant for the fundamental driver ω and the corresponding permittivity even approaches zero at the highest fluence, meaning that photocarrier injection can tune ZnO into the ENZ regime, similar to common methods, such as elemental doping.

Moreover, the transient ENZ effect leads to laser field enhancement at the probe wavelength and subsequently increases the nonlinear polarization and to an increased Fresnel reflection efficiency. Taking these two effects into account, we calculate the signal changes of HG due to reflectivity and nonlinear polarization changes in Figure 3b. The magnification of HG signals increases with the prepulse fluence, consistent with the experimental observations in Figure 1. Moreover, the dependence on the driving wavelengths is also accurately captured by our model calculation: the longer the driving wavelength is, the larger the HHG enhancement will be. This is a direct result of the ENZ effect: free carriers excited by the prepulse can tune the ENZ regime where the real part of permittivity is zero, from mid- to near-infrared ranges. In addition, the HHG enhancements of the p- and s-polarized driving beams are calculated and plotted as a function of the prepulse fluence in Figure 3c. The larger enhancement of HHG from the p polarization is one of the intrinsic properties of the ENZ effect because the laser field can be amplified only when propagating through the air-crystal interface, i.e., in p-polarization. It should be noted that the HHG signals generated by the s-polarized laser are enhanced as well albeit less strongly, which can be assigned to the contribution of the Fresnel reflection coefficient.

**Figure 4:**
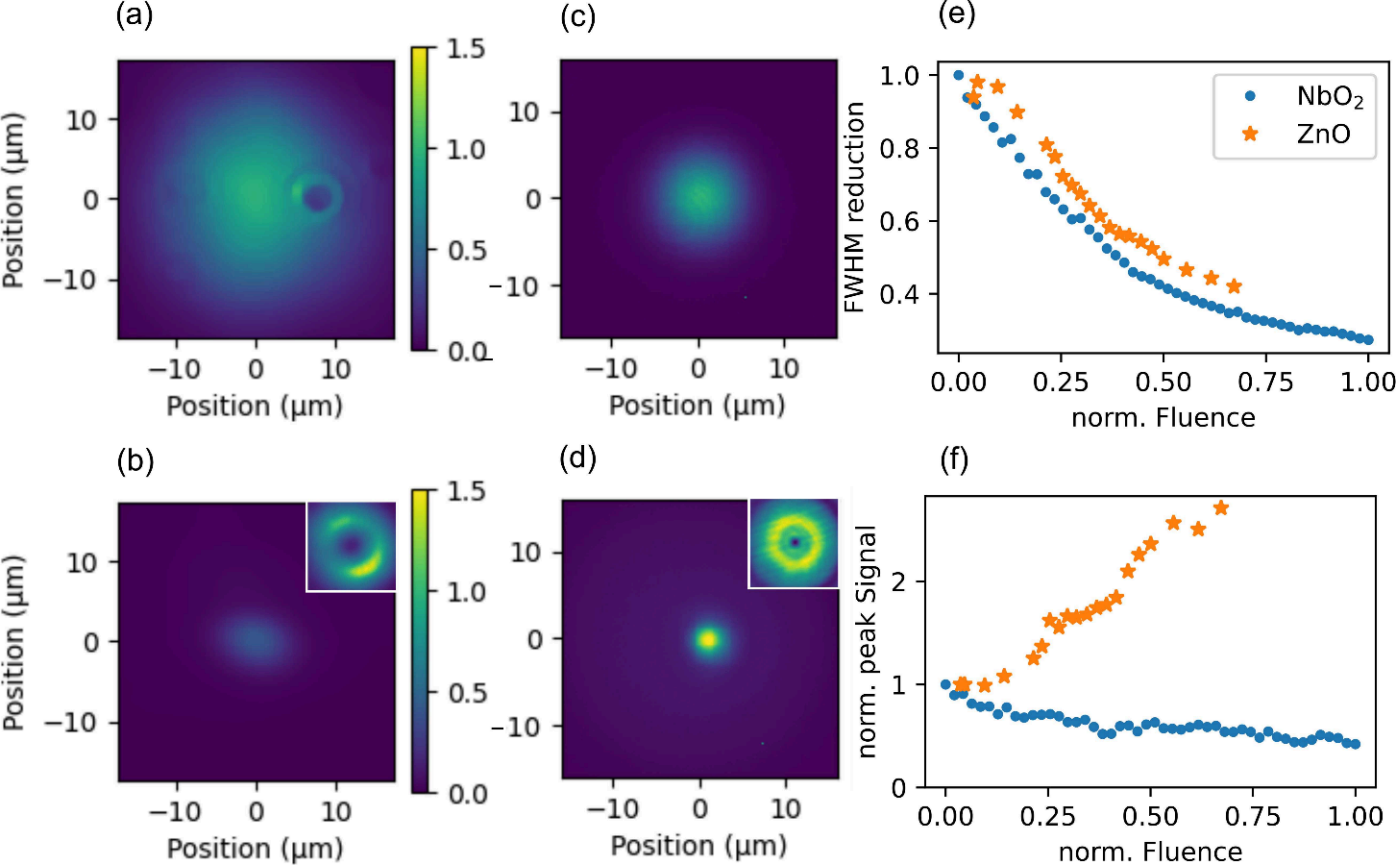
(a, c) Intrinsic microscopy images of HO3 from NbO_2_ and ZnO, respectively. (b, d) Microscopy images for HO3 reduced by a donut-shaped photocarrier injection pulse (insets) for NbO_2_ and ZnO, respectively. (e) Reduction of fwhm of the harmonic emission profile as a function of the fluence of the donut-shaped prepulse. (f) Increase/decrease of peak (central) emission strength as a function of the fluence of the donut-shaped prepulse for ZnO and NbO_2_, respectively. The fluence in (e,f) is normalized to the maximum applicable fluence in NbO_2_ before sample damage sets in, which was found to be 30 mJ/cm^2^ in ref (30).

The model calculation in Figure 3 therefore validates all experimental observations in Figures 1 and 2 and proves the hypothesis of a transient ENZ effect that enhances HG. We now propose and demonstrate an application of this ENZ effect for engineering point-spread functions (PSFs) that have a use case in femtosecond label-free super-resolution microscopy. Recently, we demonstrated that the spatial profile of harmonic generation (a microscopy image of HO3 of NbO_2_ shown in Figure 4a) could be deactivated by a donut-shaped pump pulse in focus (inset of Figure 4b), which leads to a reduction of the full width at half-maximum (fwhm) of the spatial profile (Figure 4b) well below the Abbe diffraction limit.^39^ This reduced PSF can be used in a scanning microscope for super-resolution imaging, named harmonic deactivation microscopy (HADES). Typically, due to this reduction of the emission area, the overall light emission is reduced in HADES. In other words, resolution comes at the expense of the flux reduction of emitted photons. This is visible in Figure 4b.

For the case of ZnO, the transient ENZ effect provides an opportunity to overcome this disadvantage. The microscopic emission profiles of HO3 from ZnO without and with a donut-shaped prepulse (inset of Figure 4d), are displayed in Figure 4c and 4d. We observed a similar reduction in the fwhm of the PSF just as in NbO_2_ (Figure 4b), but with the notable difference that the peak emission in the center of the PSF (Figure 4d) is increased compared to that in the scheme without a prepulse (Figure 4c). This is caused by the ENZ effect that, if pumped by a donut prepulse, creates a transient lens, i.e. a refractive index profile that follows the intensity envelope of the control pulse, with a minimum (ENZ effect) at the maximum of the donut fluence. This reshaping of the refractive index focuses the HG driving pulse further and acts as a waveguide. Instead of only suppressing HG, pumping with a donut-shaped prepulse now actually leads to a concentration of the driving electric field, which enhances HG in the center. We analyze this effect further in Figures 4e and 4f. In Figure 4e we plotted the fwhm of the PSF of NbO_2_ and ZnO, which shows a similar reduction for both cases. In Figure 4f we follow the peak emission for HO3 in NbO_2_ and ZnO, which clearly shows the enhancement present for ZnO, whereas NbO_2_ has a slight reduction that can be attributed to little remaining intensity in the center of the prepulse. These results show that not only is super-resolution microscopy (HADES) in ZnO via HG possible, but also that the PSF reduction in ZnO does not come at the expense of emitted photons.

## Conclusion

In this letter, we reported a broadband and giant enhancement of nonlinear optical signals through photoinduced permittivity engineering. Transient reduction of permittivity, due to the photoexcited carriers, can boost HG signals in ZnO by more than 2 orders of magnitude. Our results provide the opportunity to customize materials with larger nonlinearity for nanophotonics. One concrete application of this effect was also demonstrated, where the permittivity engineering created a transient waveguide that helps reduce the width of a PSF, but increases its emission strength at the center.

## Methods

### Experimental Setup

In our experimental measurements, a commercial Ti:sapphire laser amplification system (Astrella from Coherent) works as a laser source, which outputs a 35 fs pulse with a central wavelength of 800 nm. The 6-mJ laser pulse is divided into two parts, and the majority is used to pump the optical parametric amplifier (OPA). The near-infrared pulse from the X-ray laser of the OPA, ranging from 1500 to 2400 nm, is focused onto the ZnO crystal by a lens of *f* = 20 cm and generates HG signals. The reflected HG signals are collected and refocused into the fiber spectrometer (Avantes) through a pair of lenses. Another part of the fundamental laser source is frequency doubled to 400 nm and delay-controlled with a motorized translation stage (Physik Instrumente) to work as the prepulse beam for photocarrier injection. Intensity control of both beams is realized through wave plates and polarizers, whereas their polarizations are kept fixed at p-polarization for all measurements except the polarization-dependent tests. The angles of incidence for the prepulse and driving pulse are 30 and 50 degrees respectively, and the corresponding focus spot sizes of 250 and 45 μm (full width at half-maximum in intensity)ensure homogeneous excitation within the probed volume. An optical shutter (Vincent Associates) is also installed in the prepulse beam to measure HG signals with and without the prepulse.

### Model to Calculate HHG Enhancement

According to the Drude model, the density and effective mass of electrons can strongly change the permittivity of materials:
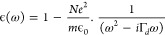
1where *N*, *e*, *m*, and Γ_*d*_ are the density, charge, effective mass, and damping rate of electrons. ϵ_0_ is the vacuum permittivity. Thus, free carriers excited by the prepulse in our experiment could play a significant role in determining the transient permittivity of ZnO. In a previous paper, Saha et al. managed to extract the transient permittivity of ZnO thin films under various photocarrier injection fluences by analyzing the transient linear reflection.^26^

In principle, HHG in the specular reflection originates from a dipolar nonlinear polarization *P*_*NL*_ of the bulk generated by the driving pulse, which is localized in a thin layer near the surface, roughly a harmonic-wavelength thickness.^43^

The transient electric field of the fundamental inside the material must be calculated first because this directly defines the nonlinear polarization strength. With the increase of the excitation fluence, the permittivity of ZnO (specifically the real part) can be reduced to zero and goes into the epsilon-near-zero (ENZ) regime, shown in Figure 3a. In this regime, a small change of permittivity could lead to a giant enhancement of the electric field of the fundamental in the crystal due to the continuity of the electric displacement at the interface without a surface charge. For a *p*-polarized laser field *E*_0_ with a given angle of incidence θ, the total field within the crystal can be expressed as
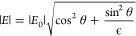
2where *E*_0_ and ϵ represent the electric field in air and the permittivity of the crystal. Therefore, the electric field *E* within an ENZ medium can be much larger than the incident field, and this enhancement strongly depends on the incident angle and permittivity change. Moreover, it can be known from the above relation that this enhancement can be observed only at a *p*-polarized laser.

Second, Fresnel reflection equations also suggest that the amplitude of the reflected wave is linked to that of the polarization by nonlinear reflection coefficients *r*_*NL*_:
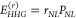
3where *P*_*NL*_ represents the nonlinear polarization, defined by the classical perturbative theory, and depends on the laser field and nonlinear susceptibility of materials. In optically isotropic materials, the nonlinear reflection coefficients for a *p*-polarized light are calculated as
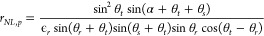
4where ϵ_*r*_ is the relative permittivity of the crystal, α is the angle between the polarization and the k-vector of *P*_*NL*_ (α = 90° in our case), θ_*r*_ is the propagation angle of the reflected wave (obtained by the conservation of momentum), θ_*t*_ is the propagation angle of the harmonic wave transmitted in the crystal, and θ_*s*_ is the propagation angle of the polarization in the crystal. These angles can be calculated from Snell’s law.
